# Coexpression of TRPML1 and TRPML2 Mucolipin Channels Affects the Survival of Glioblastoma Patients

**DOI:** 10.3390/ijms23147741

**Published:** 2022-07-13

**Authors:** Giorgio Santoni, Federica Maggi, Consuelo Amantini, Antonietta Arcella, Oliviero Marinelli, Massimo Nabissi, Matteo Santoni, Maria Beatrice Morelli

**Affiliations:** 1 School of Pharmacy, University of Camerino, 62032 Camerino, Italy; oliviero.marinelli@unicam.it (O.M.); massimo.nabissi@unicam.it (M.N.); mariabeatrice.morelli@unicam.it (M.B.M.); 2 School of Biosciences and Veterinary Medicine, University of Camerino, 62032 Camerino, Italy; federica.maggi@unicam.it (F.M.); consuelo.amantini@unicam.it (C.A.); 3 Neuropathology Laboratory, Istituto di Ricovero e Cura a Carattere Scientifico Neuromed, 86077 Pozzilli, Italy; arcella@neuromed.it; 4 Medical Oncology Unit, Hospital of Macerata, 62100 Macerata, Italy; mattymo@alice.it

**Keywords:** glioblastoma, heterogeneity, mucolipin channels, overall survival

## Abstract

Among brain cancers, glioblastoma (GBM) is the most malignant glioma with an extremely poor prognosis. It is characterized by high cell heterogeneity, which can be linked to its high malignancy. We have previously demonstrated that TRPML1 channels affect the OS of GBM patients. Herein, by RT-PCR, FACS and Western blot, we demonstrated that TRPML1 and TRPML2 channels are differently expressed in GBM patients and cell lines. Moreover, these channels partially colocalized in ER and lysosomal compartments in GBM cell lines, as evaluated by confocal analysis. Interestingly, the silencing of TRPML1 or TRPML2 by RNA interference results in the decrease in the other receptor at protein level. Moreover, the double knockdown of TRPML1 and TRPML2 leads to increased GBM cell survival with respect to single-channel-silenced cells, and improves migration and invasion ability of U251 cells. Finally, the Kaplan–Meier survival analysis demonstrated that patients with high TRPML2 expression in absence of TRPML1 expression strongly correlates with short OS, whereas high TRPML1 associated with low TRPML2 mRNA expression correlates with longer OS in GBM patients. The worst OS in GBM patients is associated with the loss of both TRPML1 and TRPML2 channels.

## 1. Introduction

Malignant glioblastoma (GBM) remains the deadliest human brain tumor with a poor prognosis despite years of research in anti-tumoral therapeutics strategies [1]. A hallmark characteristic of GBMs is their molecular and cellular heterogeneity, which is considered one of the reasons for their high malignancy and recurrence [2,3,4].

The transient receptor potential mucolipin (TRPML) channels belong to the TRP superfamily of ion channels [5]. The endolysosomal TRPML subfamily consists of TRPML1, TRPML2 and TRPML3 proteins codified by three *MCOLN1*, *MCOLN2* and *MCOLN3* genes. Several recent reports have clearly evidenced an emerging role for the TRPML channels in cancer development and progression and a clinical prognostic role was suggested [6,7,8,9].

Thus, TRPML1 overexpression has been found in cancer-bearing, oncogenic HRAS mutations [10], triple-negative breast cancer (TNBC) cell lines [11], melanomas [12], non-small-cell lung carcinomas (NSCLCs) [13], pancreatic adenocarcinomas (PDACs) [14] and p53-deficient bladder cancer [15]. Increased TRPML2 expression in HN31 oral cancer cells [10] and downregulation of the *MCOLN2* gene, due to DNA methylation, in pediatric acute lymphoblastic leukemia [16], lung adenocarcinoma and squamous cell carcinoma [17] were reported. In addition, the Human Protein Atlas reports the expression of TRPML2 mRNA and proteins in colon glandular cells and in colorectal (CRC) cancers; a significant relationship between CDH1 rs9929218 polymorphism, decreased TRPML2 expression and CRC susceptibility was demonstrated [18]. In breast cancer, Huang et al. identified a 16-gene signature, including the Wnt/β-catenin signaling pathway and the *MCOLN2* gene, associated with clinical ER and HER2 phenotype and recurrence, metastasis and distinct survival patterns [19]. Moreover, a role for TRPML2 in prostate cancer progression via the IL-1β/NF-κB pathway was recently reported [20]. *MCOLN3* is comprised in a nine-gene signature established to predict overall survival of pancreatic cancer [21].

In regard to the expression and role of TRPML channels in gliomas, we previously reported that TRPML2 silencing inhibits cell viability and proliferation [22] and increases the invasion [23] in T98 and U251 GBM cells; activation of TRPML1 channels by the MK6-83 agonist inhibits cell viability and induces apoptosis in T98 and U251 cells [24] and silencing of TRPML1 promotes the survival and invasion capability in T98 and U251 cells by affecting the autophagy and cell senescence processes [25]. Finally, concordantly with the Human Protein Atlas showing no TRPML3 mRNA expression in GBM biopsies, no TRPML3 transcripts were evidenced in the T98 and U251 cell lines [24].

The TRPML channel-mediated functions are essential in the maintenance of lysosome integrity and, consequently, a role in the regulation of cell viability was reported [26,27]. Mutations of human TRPML1 cause the mucolipidosis type IV (MLIV) neurodegenerative disease. In addition, the TRPML2 channel activity, which regulates the intracellular Ca^2+^ trafficking, is able to influence cell viability [26].

Subcellular localization studies on human TRPML proteins suggest that both the TRPML1 and TRPML2 channels may play a role in endocytic and exocytic signaling pathways [28,29,30,31]. They are located mainly in lysosomal and endosomal vesicles, although in GBM cells, TRPML1 can also be present in the nucleoplasm [24]. Moreover, it was previously reported that the loss of functional TRPML channels may influence the expression of each of the other TRPML channels [32].

After gathering together this evidence, we investigated the distribution of TRPML1 and TRPML2 channels and the mechanisms regulating their expression in GBM patients and cell lines. Moreover, the in vivo effects of the TRPML1 and TRPML2 channels’ co-expression in the survival of GBM patients by Kaplan–Meier analysis were studied. Finally, the oncogenic potential of the TRPML1 and TRPML2 double-negative (DN) subpopulations in GBM cell lines was evaluated.

## 2. Results

### 2.1. Low TRPML2 mRNA Expression Enhances the Overall Survival (OS) of GBM Patients

**Figure 1:**
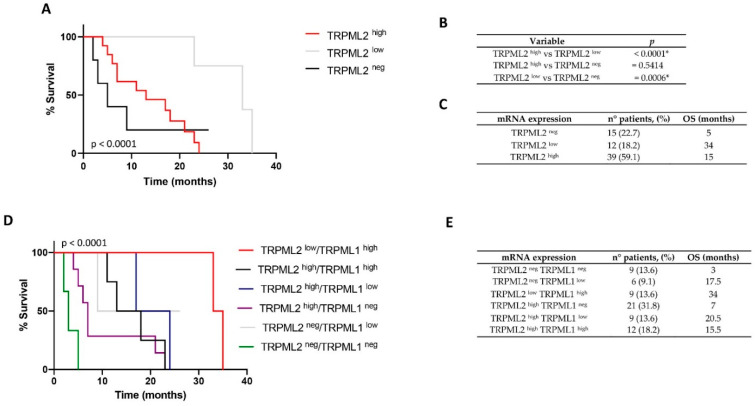
Survival analysis by Kaplan–Meier curves and log-rank (Mantel–Cox) test according to different TRPML1/TRPML2 phenotypes of GBM patients. (**A**) Stratification of GBM patients based on TRPML2 mRNA expression. (**B**) *p*-values related to Kaplan–Meier analysis. * *p* < 0.05 was considered statistically significant. (**C**) OS of patients stratified by negative, low- or high-expressing TRPML1 or TRPML2. Percentages represent the number of patients in each subgroup. (**D**) Stratification of GBM patients based on negative, low or high mRNA expression of both TRPML1 and TRPML2. (**E**) OS of patients stratified in the six subgroups. Percentages represent the number of patients in each subgroup. Neg, negative; pos, positive; high, high-expressing; low, low-expressing.

The expression of TRPML2 mRNA was evaluated in 66 GBM patients (Table S1) and OS was evaluated by Kaplan–Meier analysis. RT-PCR analysis evidenced about 77.3% of GBM patients as TRPML2-positive, whereas the other 22.7% were negative. ROC analysis was used to stratify TRPML2-positive patients as high- or low-expressing; then, OS was evaluated by Kaplan–Meier analysis. The low TRPML2 expression was significantly correlated with the higher survival time of GBM patients, whereas negative and high-expressing GBM patients were associated with a reduction of OS compared to low-TRPML2-expressing GBM patients (Figure 1A–C).

### 2.2. Correlation of TRPML1 and TRPML2 mRNA Expression with OS of GBM Patients

Regarding TRPML1, we previously reported that 54.4% of GBM patients are positive, whereas 45.6% are negative [24]. The Kaplan–Meier curves, based on the ROC analysis that stratified TRPML1-positive patients as high- and low-expressing TRPML1, displayed that TRPML1-negative patients have a poor prognosis (OS 5.5 months), whereas high-TRPML1-expressing patients have the better prognosis (OS 28 months) with respect to low-TRPML1-expressing patients (OS 17 months).

However, at present, data on the co-expression of both channels in GBM patients as well as its consequence on the survival of GBM patients have not been approached thus far. Herein, the effect of the co-expression of both TRPML2 and TRPML1 mRNA on OS was evaluated.

By crossing the high, low or negative TRPML1 and TRPML2 mRNA expressions evaluated simultaneously in each GBM specimen, out of the potential nine subgroups, no TRPML2^neg^ TRPML1^high^, TRPML2^low^ TRPML1^low^ and TRPML2^low^ TRPML1^neg^ subgroups were found on the 66 GBM patients analyzed. Thus, the OS analysis was evaluated in six GBM subgroups (Figure 1D,E, Tables S2 and S3). We found that the loss of both TRPML2 and TRPML1 channels (TRPML2^neg^ TRPML1^neg^ GBM subgroup) was associated with the worst prognosis (OS 3 months), followed by the loss of TRPML1 expression in high-TRPML2-expressing GBM patients (TRPML2^high^ TRPML1^neg^ (OS 7 months)). The GBM patients with the better prognosis were the TRPML2^low^ TRPML1^high^ subgroup (OS 34 months).

### 2.3. Coexpression of TRPML1 and TRPML2 Proteins inT98 and U251 Cells

**Figure 2:**
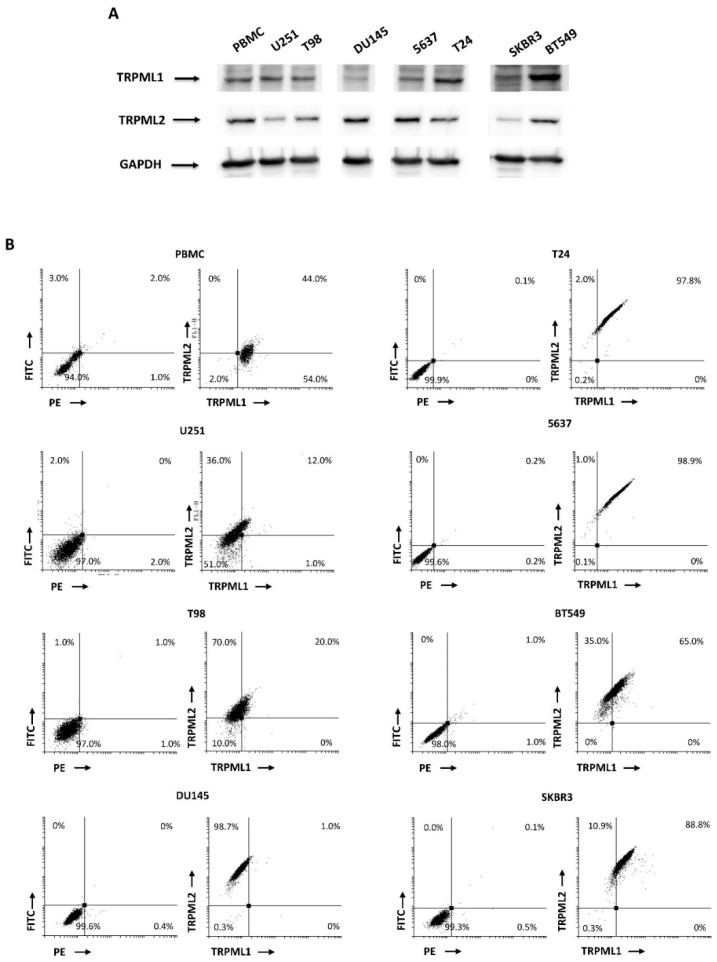
TRPML1 and TRPML2 expression in cancer cell lines. (**A**) Representative immunoblots reflecting TRPML1 and TRPML2 protein levels in U251 and T98 (glioma), DU145 (prostate cancer), 5637 and T24 (bladder cancers), and SKBR3 and BT549 (breast cancers) cell lines, and PBMCs used as positive control. Blots are representative of one of three separate experiments. (**B**) Representative FACS dot plots of TRPML1 and TRPML2 expression in U251, T98, T24, 5637, DU145, SKBR3 and BT549 cell lines and PBMCs. Plots with FITC and PE as labels represent the staining of cells with secondary Abs alone. Percentages refer to the totality (100%) of cells.

Several authors reported the expression and the role of TRPML1 and TRPML2 channels in different cancer cell lines and tissues [33,34]; however, at present, no data on their potential co-expression in cancer cell lines and tissues have been provided. Thus, we firstly evaluated the expression of the TRPML1 and TRPML2 channels by Western blot analysis in T98 and U251 GBM, DU145 prostate cancer, 5637 and T24 bladder cancer, and SKBR3 and BT549 breast cancer cell lines, and in the peripheral blood mononuclear cells (PBMCs) used as positive control (Figure 2A). Then, the co-expression of both TRPML1 and TRPML2 channels was evaluated by cytofluorimetric analysis. Of the results obtained at mRNA levels in the biopsies of GBM patients, three distinct subpopulations were evidenced in the T98 and U251 cell lines: TRPML2-positive/TRPML1-negative, TRPML1- and TRPML2-positive, and TRPML1- and TRPML2-negative (Figure 2B). TRPML2-positive but TRPML1-negative, and TRPML1- and TRPML2-positive cell populations were also evident in T24 and 5637 bladder cancer and BT549 and SKBR3 breast cancer cell lines, whereas TRPML1- and TRPML2-positive, and TRPML1-positive but TRPML2-negative cells were evident in normal human peripheral blood mononuclear cells (PBMCs) used as positive control. The DU145 prostate cancer cell line expresses only the TRPML2 channel.

### 2.4. Subcellular Localization of the TRPML1 and TRPML2 Channels in GBM Cell Lines

**Figure 3:**
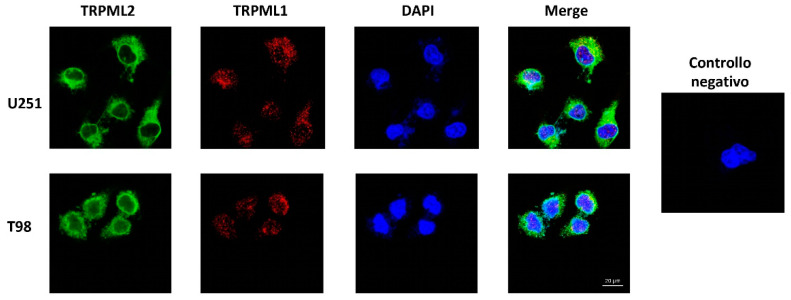
Subcellular distribution of TRPML1 and TRPML2 in glioma cell lines. Confocal microscopy analysis of TRPML1 and TRPML2 in U251 and T98 cell lines. 40,6-diamidino-2-phenylindole (DAPI) was used to counterstain nuclei. Magnification 60×.

The interactions between native TRPML1 and TRPML2 channels have been well documented [27,31]. In this regard, we examined the subcellular distribution of TRPML1 and TRPML2 in GBM cell lines by confocal microscopy analysis. As shown in Figure 3, by double staining, we found that TRPML1 localizes mainly in the nucleus and TRPML2 is expressed mainly in the cytoplasm. In addition, a partial TRPML1 and TRPML2 channel colocalization was observed.

**Figure 4:**
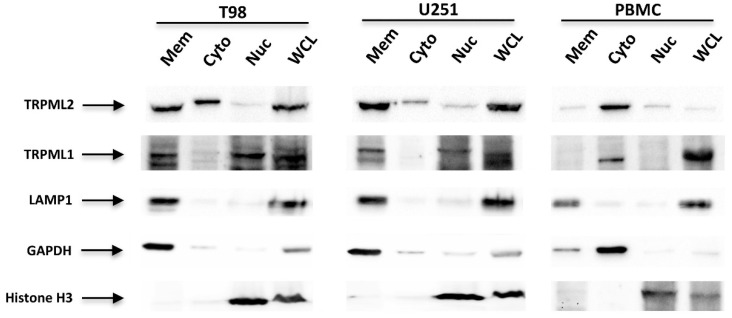
Subcellular distribution of TRPML1 and TRPML2 in glioma cell lines. Total membrane fraction (Mem), cytoplasm (Cyto), and nuclear (Nuc) extracts from T98, U251 and PMBCs, used as positive control, were immunoblotted with anti-TRPML1, anti-TRPML2, anti-LAMP1, anti-GAPDH and anti-histone H3 Ab. Whole-cell lysate (WCL) was used as control. The purity of subcellular fractions was assessed by blotting against specific markers. Cytosolic and membrane marker: GAPDH; membrane-bound organelles: LAMP1; nuclear marker: histone H3. Blots are representative of three separate experiments.

**Figure 5:**
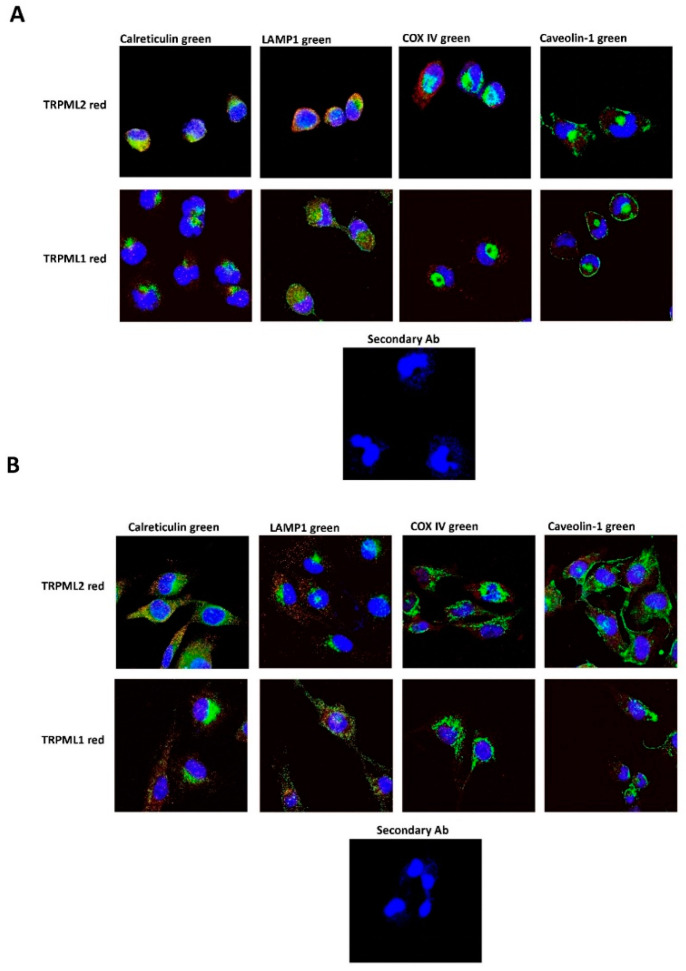
Confocal microscopy analysis to evaluate colocalization of TRPML1 or TRPML2 with different cellular organelles in T98 (**A**) and U251 (**B**) cells using specific Abs. Endoplasmic reticulum (ER): anti-calreticulin Ab; plasma membrane: anti-caveolin-1 Ab; lysosome: anti-LAMP1; mitochondria: anti-COX IV. 40,6-diamidino-2-phenylindole (DAPI) was used to counterstain nuclei. Magnification 60×.

Thus, to deepen this data, TRPML1 and TRPML2 subcellular localization was evaluated in the membrane, cytosolic and nuclear fractions by Western blot analysis. Whole-cell lysate (WCL) was used as control; LAMP1, GAPDH and histone H3 were used as markers of subcellular fractions. The TRPML2 protein was expressed in the cytoplasm and membrane fractions, whereas the TRPML1 protein localized in the nuclear and membrane fractions as previously reported [24]. Human PBMCs, used as control, evidenced a cytoplasmic localization for both the TRPML1 and TRPML2 channels (Figure 4). Moreover, the pattern of subcellular TRPML1 and TRPML2 localization was compared to the distribution of the endoplasmic reticulum (ER) (calreticulin-positive), lysosome (LAMP1-positive), mitochondria (COX IV-positive), and plasma membrane (caveolin-1-positive) markers. Both the TRPML1 and TRPML2 proteins seemed to be co-expressed in lysosome and ER compartments (Figure 5A,B and Figures S1–S4 and Table S4).

### 2.5. TRPML1 and TRPML2 Coregulation in T98 and U251 Cells

Then, by Western blot analysis, we analyzed the effect of TRPML1 or TRPML2 downregulation on protein expression levels of the other channel. At first, by qRT-PCR and Western blot analysis, we evaluated the efficacy of gene silencing. TRPML1 mRNA and protein levels were decreased in cells silenced for TRPML1 (siTRPML1) with respect to control cells (siGLO) at 72 h post-transfection; TRPML2 mRNA and protein levels were decreased in cells silenced for TRPML2 (siTRPML2) with respect to siGLO.

**Figure 6:**
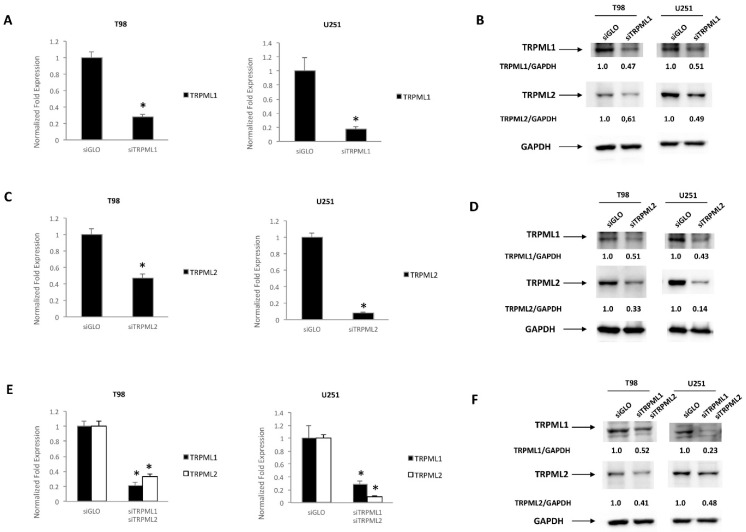
TRPML1 and TRPML2 silencing in glioma cell lines. (**A**,**C**,**E**) TRPML1 and TRPML2 mRNA levels were evaluated by qRT-PCR after 72 h of transfection in T98 and U251 cells silenced for TRPML1 (**A**), TRPML2 (**C**) or double-silenced (**E**). Relative TRPML1 and TRPML2 expression, normalized to GAPDH mRNA levels, was calculated using siGLO as calibrator. * *p* < 0.05 vs. siGLO transfected cells. (**B**,**D**,**F**) TRPML1 and TRPML2 protein levels were evaluated after 72 h of transfection in T98 and U251 cells silenced for TRPML1 (**B**), TRPML2 (**D**) or double-silenced (**F**). Blots are representative of one of three separate experiments. TRPML1 and TRPML2 densitometric values were normalized to GAPDH, used as loading control.

As shown in Figure 6, the silencing of TRPML1 (Figure 6A) results in a marked reduction of TRPML2 proteins (Figure 6B), and silencing of TRPML2 (Figure 6C) also affects the TRPML1 protein expression level (Figure 6D), suggesting a reciprocal regulation.

In double-silenced cells, a marked downregulation of both TRPML1 and TRPML2 was obtained at mRNA and protein levels (Figure 6E,F).

### 2.6. Gene Expression Profile in TRPML1/TRPML2 Double-Silenced T98 and U251 Cells

Loss of both TRPML1 and TRPML2 channels in GBM patients results in a dramatic reduction of the survival time. Thus, to evaluate the oncogenic potential of TRPML channels, the gene profile of GBM cells losing both the TRPML1/TRPML2 channels (DN) compared to wild-type (WT) T98 and U251 cells was evaluated by digital droplet PCR (Table 1). At 48 h post-transfection, increased vimentin and CD44 mesenchymal marker gene expressions were observed in DN U251, but not in DN T98 cells compared to wild-type (WT) cells. The expression of VEGFA, VEGFB, ALCAM, SPARC and NOTCH2 genes was reduced in both DN T98 and U251 cells, whereas levels of STAT3 were increased in DN U251, but not in T98 cells.

### 2.7. High Cell Growth Capability in DN Compared to siTRPML2 and siTRPML1 T98 and U251 Cells

**Figure 7:**
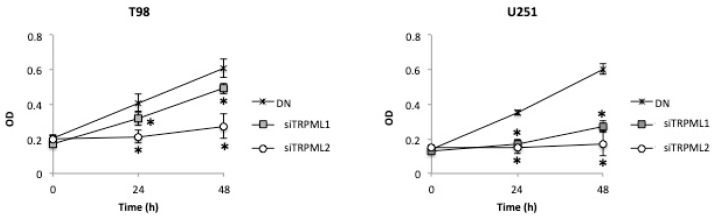
Effects of double silencing on growth of GBM cell lines. A) Cell growth was evaluated by 3-(4,5-dimethylthiazol-2-yl)-2,5-diphenyltetrazolium bromide (MTT) assay in DN, siTRPML1 and siTRPML2 T98 and U251 GBM cells for up to 48 h after silencing. Data shown are expressed as mean ± SE of three separate experiments. * *p* < 0.05 vs. siTRPML2 or siTRPML1 cells.

The TRPML1/TRPML2 DN phenotype was associated with worse OS in GBM patients. Therefore, whether the increased aggressiveness of DN GBM cells was associated with an enhanced survival of DN cells compared to single TRPML1- and TRPML2-silenced GBM cells was evaluated in vitro at 48 h after transfection by an MTT assay. A higher growth rate was evidenced in DN cells compared to siTRPML1 and siTRPML2 T98 and U251 cells (Figure 7).

### 2.8. The TRPML1/TRPML2 Double Knockdown Increases the Migration/Invasion Capability in U251 GBM Cells

**Figure 8:**
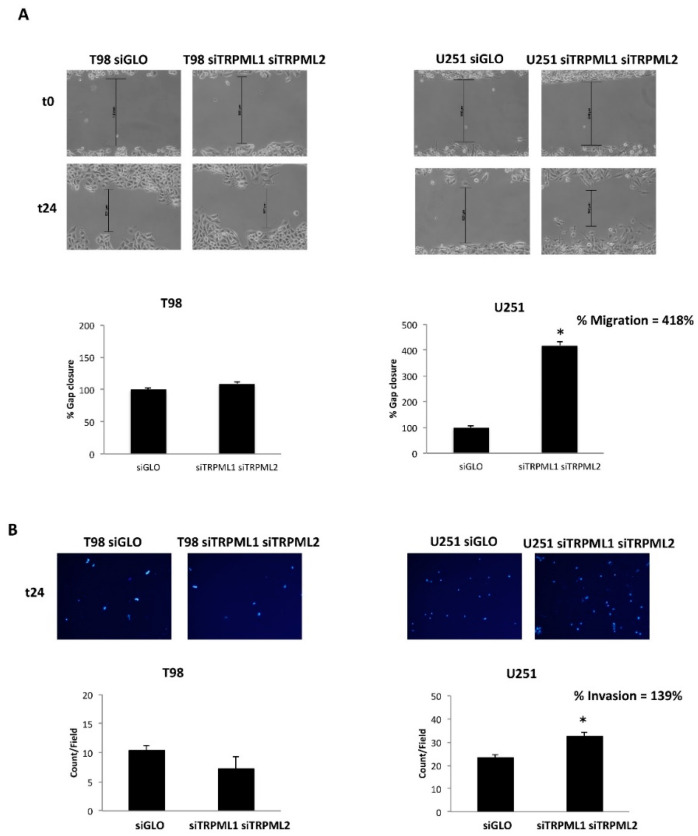
Double silencing increases the migration/invasion capability in U251 cell line. (**A**) The extent of wound closure in wound healing assays of siTRPML1 siTRPML2 (DN) T98 and U251cells at 24 h in cultures with 1% FBS. Data shown are the means ± SE. * *p* < 0.01. Representative images are shown of the progression of wound closure at 24 h. Original magnification ×10. (**B**) Representative images showing DAPI fluorescence after 24 h culture in transwell chambers (×10 magnification). The siGLO and siTRPML1 siTRPML2 T98- and U251-invading cells were counted in 10 randomly chosen microscopic fields per transwell chamber. Each sample was run in triplicate, and three independent experiments were performed. Bars represent the quantification of invaded cells in each field. Error bars represent ± SE. * *p*< 0.05.

A monolayer wound healing assay was performed to monitor the migration ability of DN (siTRPML1 siTRPML2) with respect to WT (siGLO) T98 and U251 cells. For each sample, results were reported as a percentage of wound recovery with respect to the 0 h time point. Compared with siGLO, only the migration rate of DN U251 cells significantly increased (Figure 8A).

Through the transwell invasion assay, the capability of DN vs. WT T98 and U251 cells to invade was evaluated. As shown, an increased number of invasive DAPI-positive DN U251 cells compared to WT U251 cells was evidenced by microscopic analysis. No major changes in the number of invasive DN compared to wild-type T98 cells were observed (Figure 8B).

## 3. Discussion

We previously reported that TRPML1 mRNA reduction/loss is associated with low OS and poor prognosis in GBM patients [24]; moreover, the relationship between the TRPML2 mRNA expression and pathological grading in GBM patients was also reported [22]. Here, by Kaplan–Meier analysis, we found that high TRPML2 and loss of TRPML2 mRNA expression reduce the OS and are correlated with poor prognosis in GBM patients, whereas lower TRPML2 mRNA levels are associated with better OS and clinical outcome. Biopsies from GBM patients at mRNA levels exhibit varying degrees of TRPML1 and TRPML2 expression; however, at present, no data on the influence of the co-expression or loss of each one or both of the mucolipin channels on the OS of GBM patients has been provided thus far. Among the nine GBM subgroups deriving by stratification of patients by ROC analysis based on negative, low and high TRPML2 and TRPML1 mRNA expression, six different GBM mucolipin subgroups were obtained. The Kaplan–Meier curves revealed higher OS in TRPML2^low^/TRPML1^high^ GBM patients compared to all the other GBM mucolipin subgroups. On the other hand, the complete loss of both TRPML2 and TRPML1 channels was associated with lower OS of GBM patients. The absence of TRPML1 with low- or high-expressing TRPML2 reduced the OS of GBM patients, whereas high or low TRPML1 expression showed a protective effect by increased the OS in high-expressing TRPML2 GBM patients.

The TRPML1 and TRPML2 co-expression was also demonstrated in vitro in the T98 and U251 GBM and other cancer cell lines by cytofluorimetric analysis. Moreover, by Western blot analysis we found that, as previously reported in PC-3, SK-MEL-30, U-2-OS and GBM cell lines [22,24,35], TRPML1 is mainly expressed in the nucleus, whereas TRPML2 show a cytoplasmic distribution. Moreover, TRPML1 partially colocalizes with TRPML2 in the membrane compartment. In particular, by confocal microscopy, using specific subcellular markers, we demonstrated that both TRPML1 and TRPML2 proteins localize in ER and lysosomal compartments, leaving open the possibility of heterodimerization. In this regard, subcellular localization studies of heterologously expressed mouse or human TRPML proteins have suggested that all three members can interact with each other (multimerization) [30,31]. However, endogenous TRPML subunits only partially colocalize within lysosomal and extralysosomal compartments [36], suggesting that each TRPML protein likely exists as a mostly homomeric channel.

It was previously reported that the loss of a functional TRPML channel may influence the expression of other TRPML channels. Significant reductions of the TRPML2sv (short isoform), but not of the TRPML2lv (long isoform) or TRPML3 transcripts, were observed in lymphoid and kidney organs of TRPML1^-^^/-^ mice [32]. By RNA interference of endogenous human TRPML1 in HEK-293 and HeLa cells, a decrease in TRPML2 transcripts that can be restored by human TRPML1 overexpression [32] was evidenced, supporting the involvement of TRPML1 in the regulation of TRPML2 expression. The TRPML2 channel is unlikely to compensate for the loss of TRPML1 and TRPML1 appears to play a novel role in the tissue-specific transcriptional regulation of TRPML2 [32]. In this regard, we demonstrated that TRPML2 silencing markedly reduces the TRPML1 protein expression in T98 and U251 cells; similarly, silencing of TRPML1 slightly reduced the TRPML2 expression levels in both cell lines. Thus, our data suggest co-regulation of TRPML channels also in GBM. The transcription of the TRPML1 gene requires a TFEB transcription factor [37], whereas transcription of TRPML2 seems to be regulated by PAX5 [38]. In dendritic cells, the activation of TRPML2 induced TFEB-dependent transcription [39] and genetic or pharmacological inhibition of TRPML1 also results in TFEB nuclear translocation that activates positive feedback with transcription of lysosomal genes, including TRPML1 [40]. Moreover, significant upregulation of TRPML2sv transcripts was observed when primary mouse lymphoid cells were treated with TRPML1 activators [36].

A hallmark characteristic of GBM is its cellular heterogeneity that is the major cause for high malignancy and low survival of GBM patients [2]. Herein, we found that loss of both the TRPML1 and TRPML2 channels in GBM cells results in the acquisition of a more aggressive phenotype. We displayed that GBM patients harboring a TRPML1 and TRPML2 DN GBM phenotype showed a very dismal OS, compared to the other GBM patients. We also demonstrated in vitro that double TRPML2 and TRPML1 knockdown in GBM cells showed a more aggressive phenotype, mainly in U251 cells. Thus, DN GBM cells compared to siTRPML2 or siTRPML1 T98 and U251 cells showed an increased growth capability, mesenchymal stem-like phenotype, augmented STAT3 proliferative level and increased migration/invasion capability, compared to wild-type control cells. Finally, given the role of the VEGFA/NOTCH2 signaling pathway in TRPML2-positive GBM cells [23], a reduced VEGFA/B and NOTCH2 mRNA expression was detected in DN siTRPML2/TRPML1 GBM cells.

**Figure 9:**
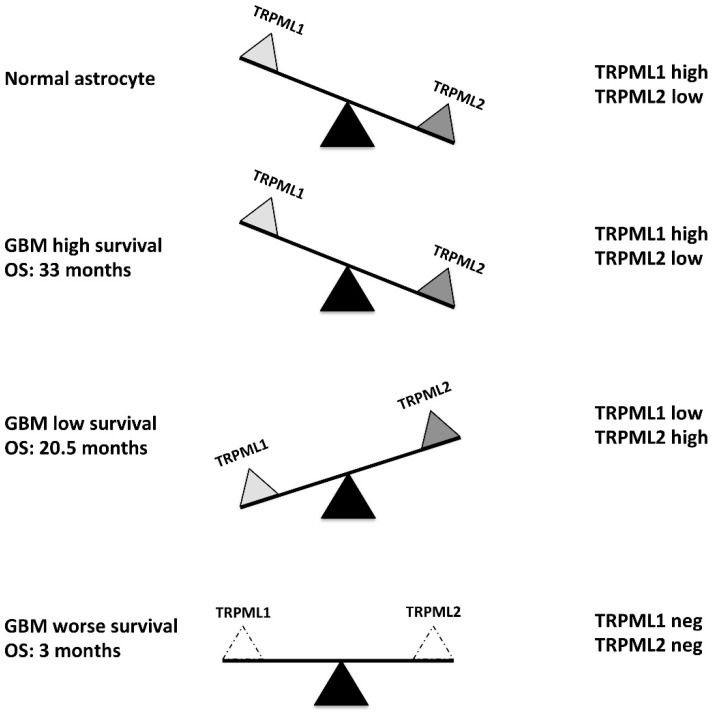
Schematic representation of TRPML1 and TRPML2 modification in GBM.

Overall, our data suggest that both TRPML1 and TRPML2 channels play a key role in the survival of GBM patients and the loss of both receptors is associated with the worst survival of GBM patients. Moreover, we found that some GBM tissues and normal human astrocytes share the same “Mucolipin channel phenotype”, TRPML1^high^/TRPML2^low^ [22,24]. Tumor cells progressively reduce TRPML1 and increase TRPML2 mRNA expression. Thus, in GBM, changes in TRPML1 and TRPML2 expression seem to be likely associated with tumor progression rather than tumor development. The cellular switch from normal astrocytes to neoplastic GBM cells is associated with a drastic reduction in the OS and a worse prognosis in the GBM patients who lose both the TRPML1 and TRPML2 channels (Figure 9). The identification of the cellular heterogeneity in GBM patients in relation to the different TRPML channel expression levels must be the upcoming task to provide significant and personalized therapeutic strategies in patients with GBM.

## 4. Materials and Methods

### 4.1. Cells and Tissues

Formalin-fixed, paraffin-embedded brain tissues from human tumor biopsies and epileptic brains (EHBs) (n = 2) surgically removed from patients who gave informed consent to the study (n = 66) were kindly provided by Prof. Antonietta Arcella (I.N.M., Neuromed, Pozzilli, Isernia, Italy). Glioblastoma tissues (grade IV) were histologically graded according to the World Health Organization’s classification criteria. Informed consent was obtained before surgery according to the Neuromed Ethics Committee’s guidelines. The glioblastoma T98 and U251 cell lines, obtained from the European Collection of Cell Cultures (ECACC, Salisbury, UK), were maintained in Eagle’s minimum essential medium (EMEM, Lonza Bioresearch, Basel, Switzerland) supplemented with 10% heat-inactivated fetal bovine serum (FBS), 2 mmol/L L-glutamine, 100 IU/mL penicillin and 100 μg streptomycin.

### 4.2. Chemical and Reagents

3-(4,5-dimethylthiazol-diphenyltetrazolium bromide (MTT), was purchased from Sigma Aldrich (Milan, Italy). The following rabbit polyclonal antibodies (Abs) were used: Anti-histone H3 (1:1000; Cell Signaling Technology, Danvers, MA, USA), anti-COX IV (1:250; Cell Signaling Technology) and anti-caveolin-1 (1:400; Cell Signaling Technology). The following mouse monoclonal Abs were used: anti-TRPML1 (clone F-10, 1:300 for Western blot, 1:50 for FACS analysis; Santa Cruz Biotechnology, Dallas, TX, USA), anti-TRPML2 (1:300 for Western blot; Santa Cruz Biotechnology), anti-LAMP1 (1:300; Santa Cruz Biotechnology), anti-calreticulin (1:1000; BD Biosciences, Milan, Italy) and anti-glyceraldehyde-3-phosphate dehydrogenase (anti-GAPDH, 14C10, 1:1000; Cell Signaling Technology). Anti-TRPML2 from Sigma Aldrich was used diluted 1:50 for FACS analysis.

The following secondary antibodies were used: horseradish peroxidase (HRP)-conjugated anti-mouse IgG and HRP-conjugated anti-rabbit IgG (1:2000; Cell Signaling Technology), FITC-conjugated anti-rabbit Ab (BD Biosciences), PE-conjugated anti-mouse Ab (BD Biosciences, Milan, Italy), Alexa Fluor-594-conjugated anti-mouse Ab (1:100; Invitrogen, San Diego, CA, USA), Alexa Fluor-488-conjugated anti-mouse Ab (1:100; Invitrogen) and Alexa Fluor-488-conjugated anti-rabbit Ab (1:100; Invitrogen).

### 4.3. Western Blot Analysis

To obtain the whole-cell lysate, cells were lysed in a lysis buffer containing a protease inhibitor cocktail (EuroClone, Milan, Italy). Cytoplasmatic, membrane/organelle and nuclear/cytoskeletal fractions were isolated using the Cell Fractionation Kit (Cell Signaling Technology) according to the manufacturer’s instruction. Proteins were separated on 8–14% SDS polyacrylamide gel in a Mini-PROTEAN Tetra Cell system (Bio-Rad, Hercules, CA, USA). Protein transfer from the gel to a nitrocellulose membrane was carried out using the Mini Trans-Blot Turbo RTA system (Bio-Rad). Nonspecific binding sites were blocked with 5% low-fat dry milk and 2% bovine serum albumin (BSA) in phosphate-buffered saline 0.1% Tween 20 Detergent for 1 h at room temperature. Membranes were incubated overnight at 4 °C in primary Abs (anti-TRPML1, anti-TRPML2, anti-LAMP-1, anti-histone H3 and anti-GAPDH), followed by incubation for 1 h at room temperature with HRP-conjugated anti-rabbit or anti-mouse secondary Abs. The detection was performed using the LiteAblot PLUS or TURBO kits (EuroClone), and densitometric analysis was carried out via a Chemidoc using the Quantity One software (version 4.6.7, Bio-Rad). For quantification, GAPDH was used as loading control. One representative out of three independent experiments is shown in each immunoblot figure.

### 4.4. TRPML1 and/or TRPML2 Transfection Models

For silencing experiments, TRPML1 (siTRPML1), TRPML2 (siTRPML2), TRPML1 and TRPML2 (siTRPML1/TRPML2) and the siCONTROL nontargeting siRNA (siGLO, used as negative control), FlexiTube siRNA, was purchased from Qiagen (Milan, Italy). T98 and U251 cell lines were plated at a density of 1.2 × 10^5^/mL and siTRPML1 and/or siTRPML2 or siGLO (150 ng) was added to the wells, following the HiPerfect transfection reagent protocol (Qiagen). No differences were observed comparing siGLO transfected cells with untransfected cells.

### 4.5. MTT Assay

Three ×10^4^/mL siTRPML1, siTRPML2 and siTRPML1/TRPML2 GBM cells were plated in 96-well plates for 24 h and 48 h post-transfection. Then, 0.8 mg/mL of MTT was added to the samples and incubated for an additional 3 h. After the removal of medium from the wells, the formazan crystals were dissolved with 100 µL per well of DMSO and the colored solutions were read by a microtiter plate spectrophotometer (BioTek Instruments, Winooski, VT, USA). Four replicates were used for each treatment.

### 4.6. Cell Layer Wound Assays

In vitro wound assays were performed using ibidi culture-inserts (Ibidi GmbH, Martinsried, Germany) according to Shih et al. [41]. Briefly, when confluent monolayers of T98 and U251 cells were established on ibidi dishes (35 mm with high culture-insert coating), cells were washed twice with phosphate-buffered saline (PBS) to remove residual cell debris. To evaluate the effect of double silencing in wound healing behavior, silenced cells were cultured for 24 h in complete medium. Subsequently, inserts were removed to reveal a well-defined gap approximately 1000 μm wide and the complete medium was replaced with a medium with 1% FBS. Cells that had migrated into the wound area were photographed at 0 h and 24 h. The gap was then measured using ImageJ software at 10 different points along the gap and averaged to provide the mean gap distance. The relative distance of cell migration was calculated as:

% Gap closure = DN (Gap-t0 − Gap-t24)/siGLO (Gap-t0 − Gap-t24) × 100

where Gap-t0 is the mean gap distance prior to cellular migration immediately following the removal of the ibidi culture-insert (zero h) and Gap-t24 is the mean gap distance at the 24 h endpoint. The results are expressed as a percentage of siGLO.

### 4.7. Invasion Assay

Cell invasion was evaluated via a transwell assay using the transwell chambers (BD Biosciences, Franklin Lakes, NJ, USA) as previously described [23]. Briefly, a total of 750 μL cell culture medium supplemented with 10% FBS was added in the lower chamber. A serum-free culture medium (500 μL) containing 2.5 *×* 10^4^ glioma cells was plated into the upper chamber. After 24 h of incubation, cells remaining in the upper chamber were removed by cotton swabs. The cells were fixed in 4% paraformaldehyde and stained with DAPI. A fluorescence microscope (BX51 Fluorescence Microscope, Olympus, Milan, Italy) and ImageJ software version 1.45 s (National Institutes of Health, Bethesda, MD, USA) were used for the image acquisition at x10 magnification and analysis. Ten fields were selected at random to measure the average cell coverage. The experiments were performed in triplicate at least three times independently.

### 4.8. Confocal Laser Scanning Microscopy Analysis

The T98 and U251 cells were maintained on 8-well culture slides in fresh medium, fixed and permeabilized using 2% and 4% of paraformaldehyde with 0.5% of Triton X-100 in PBS. After washes in PBS, cells were incubated with 5% of bovine serum albumin (BSA) and 0.1% of Tween 20 in PBS for 1 h at room temperature and then stained with anti-TRPML1 and/or anti-TRPML2 Abs overnight at 4 °C. Then, samples were washed with 0.3% of Triton X-100 in PBS and incubated with Alexa Fluor 594-conjugated secondary Abs for 1 h at 37 °C. In colabeling experiments, cells were stained with anti-LAMP1, anti-calreticulin, anti-COX IV and anti-caveolin-1 Abs overnight at 4 °C. Finally, samples were washed with 0.3% of Triton X-100 in PBS and incubated with Alexa Fluor 488-conjugated secondary Abs for 1 h at 37 °C. Nuclei were stained with DAPI. Slides were then analyzed with the C2 Plus confocal laser scanning microscope (Nikon Instruments, Firenze, Italy). Optimized emission detection bandwidths were configured with Zeiss Zen control software. Images were processed using NIS-Elements imaging software (Nikon Instrument, Firenze, Italy).

### 4.9. Immunofluorescence and FACS Analysis

Cells were fixed with 4% paraformaldehyde and then stained with anti-human TRPML1 and/or TRPML2 Abs or normal mouse IgG1 isotype control (Santa Cruz Biotechnology) in a permeabilization buffer (PBS, 1% FBS, 0.1% NaN_3_ and 1% saponin). After an incubation of 1 h at 4 °C, cells were then incubated with an FITC- or PE-conjugated secondary Ab and analyzed using an FACScan cytofluorimeter with CellQuest software.

### 4.10. Gene Expression Analysis

Total RNA from fixed paraffin-embedded tissue slices (5–7 μm thick) was extracted via the RNeasy^®^ FFPE Mini Kit (Qiagen, Milan, Italy). All RNA samples were eluted in the appropriate buffer and their concentration and purity were evaluated with an A260/280 nm measurement. In total, 800 ng of RNA extracted was subjected to reverse transcription in a total volume of 20 μL using the iScript kit (Bio-Rad) according to the manufacturer’s instructions. Then, 5 μL of the cDNA derived by fixed paraffin-embedded tissue was pre-amplified for 15 cycles using the SsoAdvanced PreAmp Supermix kit (Bio-Rad). One microliter of the resulting cDNA products was used as template for quantitative real-time polymerase chain reaction (qRT-PCR). Quantitative RT-PCR was performed by using the IQ5 Multicolor real-time PCR detection system (Bio-Rad). The reaction mixture contained the SsoAdvanced Universal SYBR Green Supermix (Bio-Rad). Human TRPML1, TRPML2 and GAPDH RT2 qPCR Primer Assays (Qiagen) were used. The PCR parameters were 10 min at 95 °C, followed by 40 cycles at 95 °C for 15 s and 60 °C for 40 s. All samples were assayed in triplicate in the same plate. The relative amount of target mRNA was calculated by the 2-ΔΔCt method, using GAPDH as a housekeeping gene.

RNA from U251 and T98 cell lines was extracted via the SingleShot Cell Lysis Kit (Bio-Rad) according to the protocol. Then, 800 ng of the extracted RNA were subjected to reverse transcription in a total volume of 20 μL using the iScript kit (Bio-Rad) according to the manufacturer’s instruction, and the resulting cDNA was used to pre-amplify each sample for all primers used in the gene expression analysis by using the SsoAdvanced PreAmp Kit and Assays (Bio-Rad). The ddPCR Supermix for Probes (No dUTP) (Bio-Rad) and the specific PrimePCR^TM^ ddPCR^TM^ Expression Probe Assays conjugated with FAM or HEX fluorescent dyes (the same pool used in the pre-amplification step) (Bio-Rad) were then used to perform the digital droplet PCR (ddPCR). The analyzed target genes were: ALCAM, CD44, EPCAM, VIMENTIN, SHH, DHH, IHH, PTCH1, PTCH2, ZEB1, ZEB2, SMO, VEGFA, VEGFB, NOTCH1, NOTCH2, STAT3, POU5F1B and SPARC. Results, expressed as cDNA copies/μL, were normalized to the β-actin concentration and analyzed using the QuantaSoft Software (Bio-Rad).

### 4.11. Statistical Analysis

The statistical significance was determined by a Student’s *t*-test and by ANOVA with Bonferroni’s post-test. Overall survival was defined as the interval between the date of surgery to death or last follow-up visit. Median overall survival (OS) was estimated using the Kaplan–Meier method with Rothman’s 95% confidence intervals (CIs) and compared across the groups using the log-rank test. Depending on the TRPML2 mRNA expression, the GBM patients were divided into TRPML2-positive and -negative, and the TRPML2-positive patients were further stratified as low- and high-TRPML2-expressing GBM patients according to ROC analysis. TRPML1 [24] and TRPML2 mRNA coexpression levels were then used to stratify GBM patients in 6 specific subgroups: TRPML2^neg^ TRPML1^neg^, TRPML2^neg^ TRPML1^low^, TRPML2^low^ TRPML1^high^, TRPML2^high^ TRPML1^neg^, TRPML2^high^ TRPML1^low^ and TRPML2^high^ TRPML1^high^. These groups were subjected to Kaplan–Meier survival analyses. Statistical analysis was performed with the MedCalc package (MedCalc^®^ version 16.4.3, Ostend, Belgium).

## 5. Conclusions

The TRPML1 and TRPML2 channels play a key role in the survival of both normal astrocytes and their neoplastic-derived GBM cells. The increase in GBM malignancy and the short-term OS were associated with reduced TRPML1 and increased TRPML2 expression, as well as a complete loss of both channels. Changes in TRPML1 and TRPML2 expression were associated with tumor progression but not with tumor development. The identification of mucolipin expression levels in GBM patients could permit a personalized approach to therapy in GBM patients.

## Figures and Tables

**Table 1:** Effects of TRPML1/TRPML2 double silencing in T98 and U251 cell lines.

Target Genes	WT T98		DN T98		WT U251		DN U251	
	cDNA Copies/μL	Gene/ACTB	cDNA Copies/μL	Gene/ACTB	cDNA Copies/μL	Gene/ACTB	cDNA Copies/μL	Gene/ACTB
ALCAM	826	0.11	487 *	0.06	1153	0.10	653 *	0.06
CD44	4510	0.61	2875 *	0.38	10,200	0.90	12,161 *	1.16
EPCAM	5	-	7	-	0	-	36	-
VIMENTIN	6421	0.87	3885 *	0.52	6740	0.60	8013 *	0.76
SHH	0	-	0	-	5	-	1	-
DHH	7	-	1	-	3	-	1	-
IHH	0	-	0	-	4	-	0	-
PTCH1	83	-	85	0.01	27	-	25	-
PTCH2	2	-	1	-	102	0.01	55	0.00
ZEB1	566	0.08	297 *	0.04	110	0.01	78	0.01
ZEB2	44	-	27	-	167	0.01	49	0.00
SMO	257	0.03	207	0.03	37	-	45	-
VEGFA	1506	0.20	1048 *	0.14	3440	0.30	2055 *	0.20
VEGFB	1492	0.20	1120 *	0.15	537	0.05	440	0.04
NOTCH1	34	-	26	-	381	0.03	173 *	0.01
NOTCH2	1492	0.20	484 *	0.06	2020	0.18	645 *	0.06
STAT3	1090	0.15	778 *	0.10	984	0.09	1099 *	0.10
POU5F1B	0	-	0	-	1	-	20	-
SPARC	1835	0.25	574 *	0.08	1964	0.17	1871	0.18

Data are expressed as cDNA copies/μL and normalized for ACTB, the housekeeping gene. Abbreviations: ALCAM/CD166: activated leukocyte cell adhesion molecule; EPCAM: epithelial cell adhesion molecule; SHH: sonic hedgehog; DHH: desert hedgehog; IHH: Indian hedgehog; PTCH1: patched 1; PITCH2: patched 2; ZEB1: zinc finger E-box-binding homeobox 1; ZEB2: zinc finger E-box-binding homeobox 2; SMO: smoothened frizzled class receptor; VEGFA, vascular endothelial growth factor A; VEGFB, vascular endothelial growth factor B; NOTCH1: notch receptor 1; NOTCH2: notch receptor 2; POU5F1B: POU class 5 homeobox 1B; STAT3: signal transducer and activator of transcription 3; SPARC: secreted protein acidic and rich in cysteine; ACTB: β-actin. * *p* < 0.05 vs. WT cells.

## Data Availability

The data that support the findings of this study are available from the corresponding authors upon request.

## Supplementary Materials

The following supporting information can be downloaded at: https://www.mdpi.com/article/10.3390/ijms23147741/s1.
