# Supplementary material for: Coexpression of TRPML1 and TRPML2 Mucolipin Channels Affects the Survival of Glioblastoma Patients
Source: Int J Mol Sci. 2022 Jul 13;23(14):7741. doi: 10.3390/ijms23147741 (PMC9321332; doi:10.3390/ijms23147741)
Supplement: Supplementary file 1 [file ijms-23-07741-s001.zip › ijms-1653537-supplementary.pdf]

Supplementary files

Table S1. GBM (grade IV) patient characteristics

**Patients characteristics**	
**N° sample**	**66**
**Sex**	
Men	47% (n = 31)
Women	53% (n = 35)
**Age, years**	
< 45	55% (n =36)
>45	45% (n = 30)
**GBM MGMT methylation**	
Non detected	32% (n = 21)
Methylated	36% (n = 24)
Non methylated	32% (n = 21)
**GBM Recidivated**	
Not detected	9% (n = 6)
Not recidivated	14% (n = 9)
Recidivated	77% (n = 51)
**Adjuvant therapy**	
No	13% (n= 9)
STUPP	56% (n= 37)
Fotemustine	8% (n= 5)
RT	11% (n= 7)
Other	12% (n=8)
Temozolomide	*38% (3/8)*
Fotemustine + Bevacizumab	*38% (3/8)*
Fotemustine + RT	*12% (1/8)*
Fotemustine + RT + Bevacizumab	*12% (1/8)*

Table S2. TRPML1 and TRPML2 gene expression in GBM patients evaluated by qRT-PCR.

Pz. N°	TRPML1	TRPML2	Pz. N°	TRPML1	TRPML2	Pz. N°	TRPML1	TRPML2
	mRNA expression[a]			mRNA expression[a]			mRNA expression[a]	
#1	Low	High	#23	Neg	Neg	#45	High	Low
#2	High	Low	#24	Neg	Neg	#46	Low	High
#3	High	High	#25	Neg	High	#47	High	High
#4	High	Low	#26	Low	Neg	#48	Low	High
#5	Neg	Neg	#27	High	Low	#49	Low	Neg
#6	High	Low	#28	Neg	High	#50	Low	Neg
#7	Neg	High	#29	Neg	Neg	#51	Neg	High
#8	High	High	#30	Neg	High	#52	Neg	High
#9	High	High	#31	Neg	High	#53	Low	High
#10	Neg	Neg	#32	Low	High	#54	Low	Neg
#11	Low	Neg	#33	Low	High	#55	Neg	High
#12	Neg	High	#34	Neg	High	#56	Neg	High
#13	Low	Neg	#35	Neg	Neg	#57	Low	High
#14	High	Low	#36	High	Low	#58	Neg	Neg
#15	Neg	High	#37	Neg	High	#59	High	High
#16	Neg	High	#38	Neg	High	#60	High	High
#17	High	Low	#39	High	High	#61	Neg	High
#18	Neg	High	#40	Low	High	#62	High	High
#19	Neg	High	#41	High	High	#63	Neg	High
#20	Low	High	#42	Neg	High	#64	High	High
#21	Neg	High	#43	Neg	Neg	#65	High	High
#22	Neg	Neg	#44	High	Low	#66	High	High

[a]Relative mRNA expression; Neg: Negative; Low TRPML1 ≤ 1.0, Low TRPML2 ≤ 1.8; High TRPML1 > 1.0, High TRPML2 > 1.8 evaluated by ROC analysis.

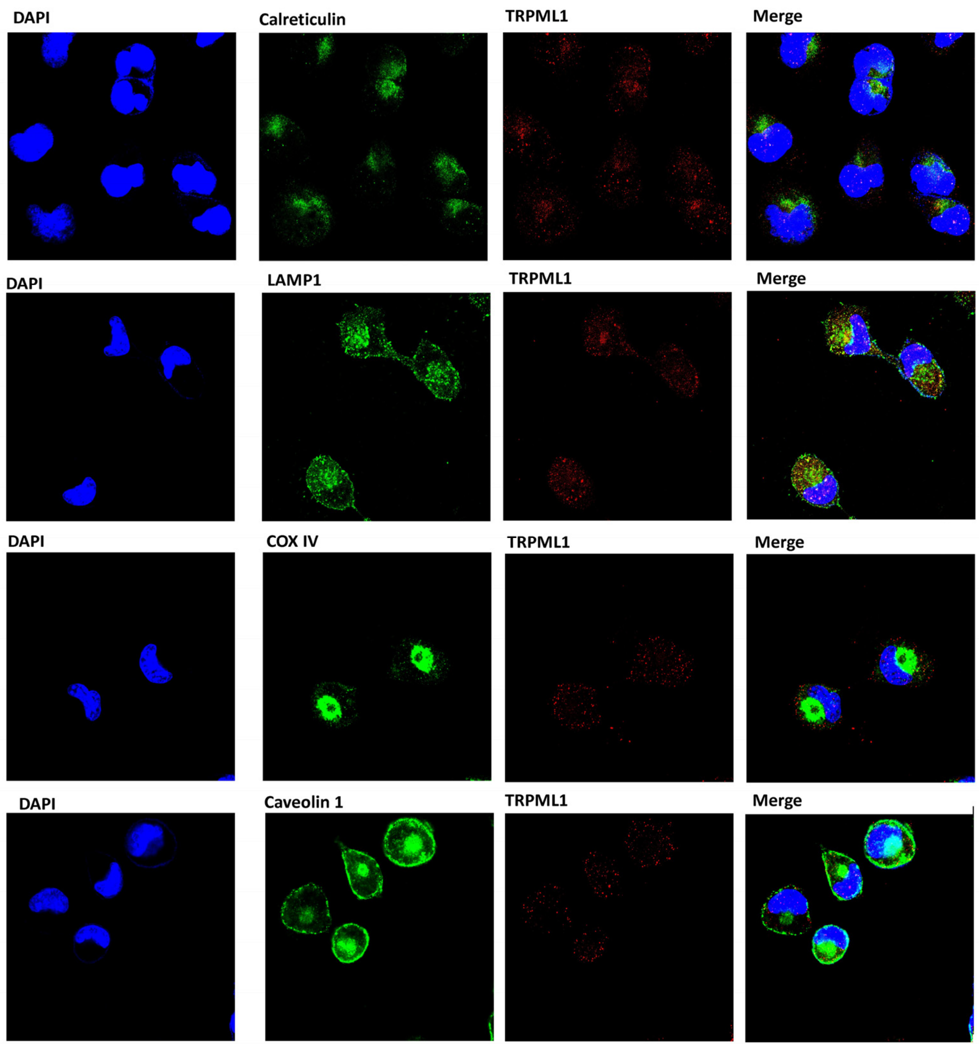

Figure S1. Confocal microscopy analysis to evaluate colocalization of TRPML1 different cellular organelles in T98 cells using specific Abs. Endoplasmic reticulum (ER): anti-calreticulin Ab; plasma membrane: anti-caveolin-1 Ab; lysosome: anti-LAMP1; mitochondria: anti-COX IV. 40,6-diamidino-2-phenylindole (DAPI) was used to counterstain nuclei. Magnification 60x.

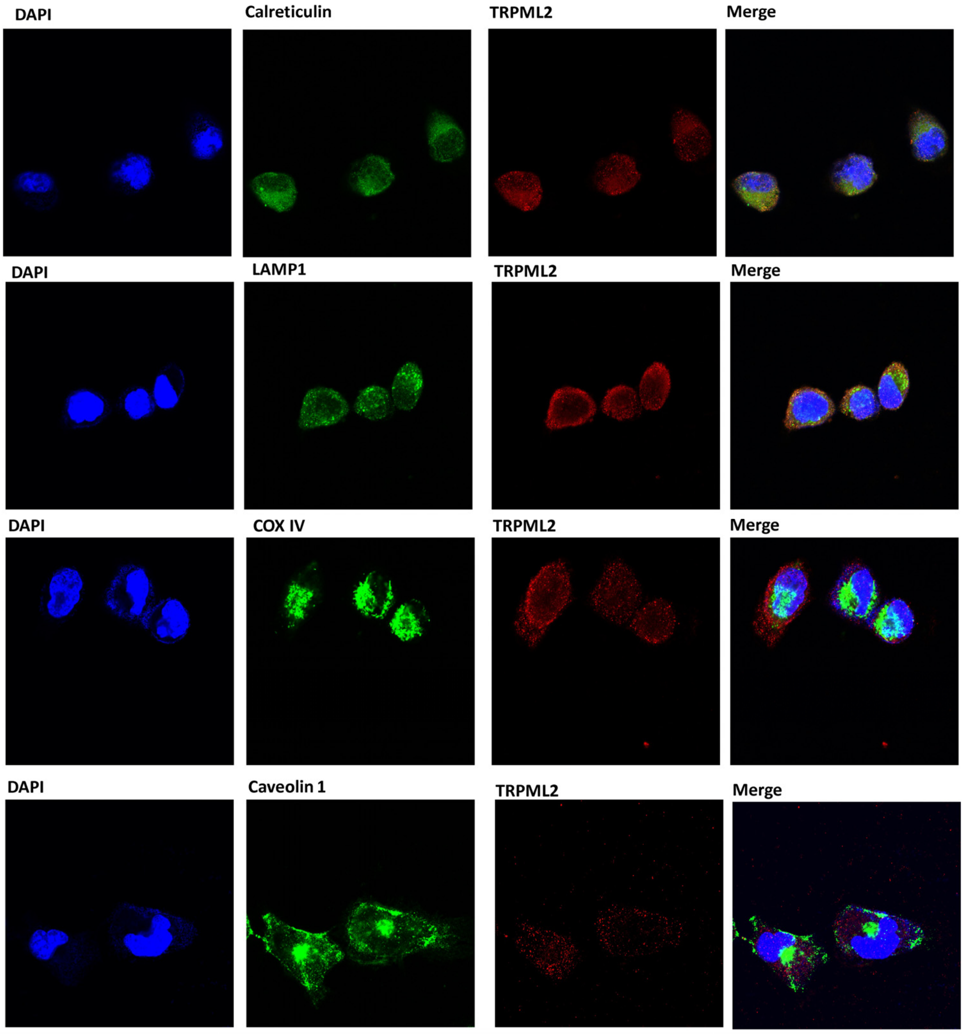

Figure S2. Confocal microscopy analysis to evaluate colocalization of TRPML2 different cellular organelles in T98 cells using specific Abs. Endoplasmic reticulum (ER): anti-calreticulin Ab; plasma membrane: anti-caveolin-1 Ab; lysosome: anti-LAMP1; mitochondria: anti-COX IV. 40,6-diamidino-2-phenylindole (DAPI) was used to counterstain nuclei. Magnification 60x.

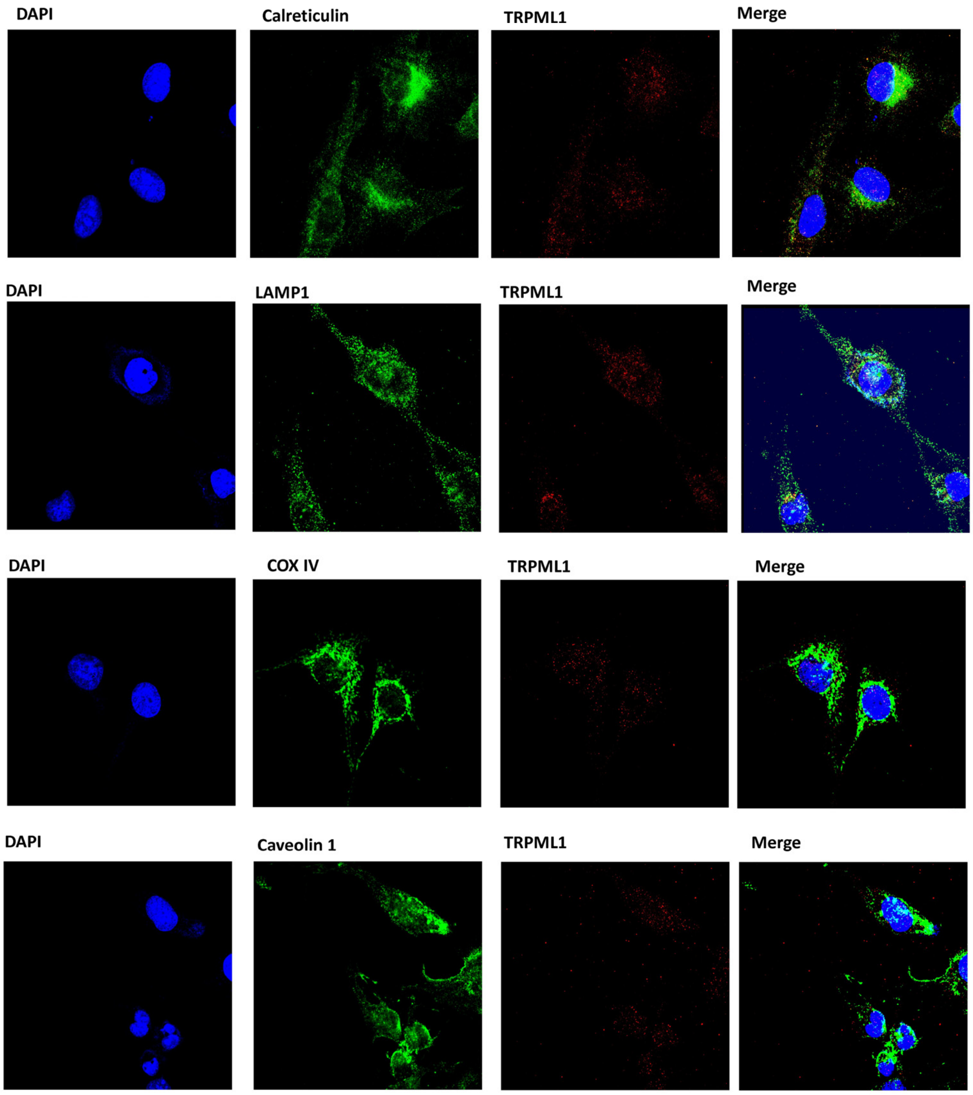

Figure S3. Confocal microscopy analysis to evaluate colocalization of TRPML1 different cellular organelles in U251 cells using specific Abs. Endoplasmic reticulum (ER): anti-calreticulin Ab; plasma membrane: anti-caveolin-1 Ab; lysosome: anti-LAMP1; mitochondria: anti-COX IV. 40,6-diamidino-2-phenylindole (DAPI) was used to counterstain nuclei. Magnification 60x.

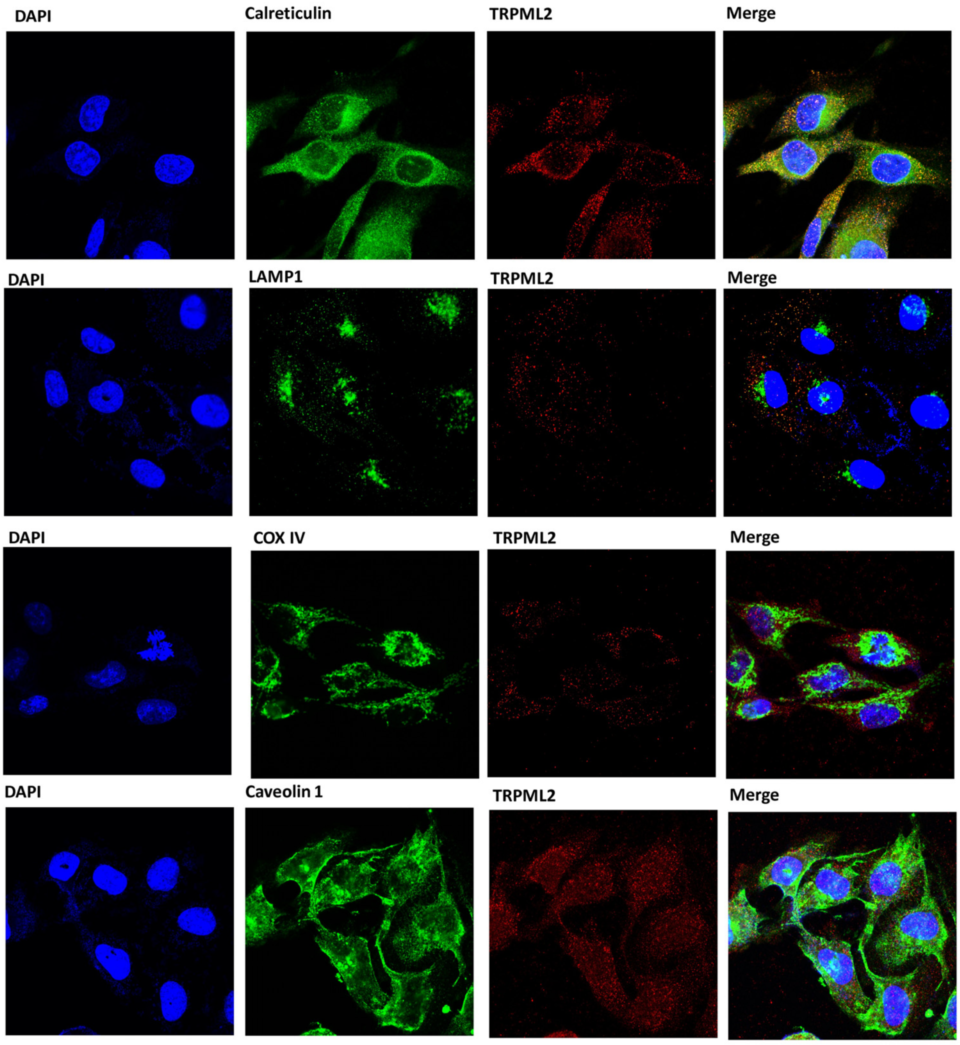

Figure S4. Confocal microscopy analysis to evaluate colocalization of TRPML2 different cellular organelles in U251 cells using specific Abs. Endoplasmic reticulum (ER): anti-calreticulin Ab; plasma membrane: anti-caveolin-1 Ab; lysosome: anti-LAMP1; mitochondria: anti-COX IV. 40,6-diamidino-2-phenylindole (DAPI) was used to counterstain nuclei. Magnification 60x.

Table S3. Kaplan-Meier p-values.

P value	TRPML2 ^{low}<br>TRPML1 ^{high}	TRPML2 ^{low}<br>TRPML1 ^{neg}	TRPML2 ^{high}<br>TRPML1 ^{high}	TRPML2 ^{high}<br>TRPML1 ^{low}	TRPML2 ^{high}<br>TRPML1 ^{neg}	TRPML2 ^{neg}<br>TRPML1 ^{neg}
**TRPML2 ^{low}<br>TRPML1 ^{high}**		0,0219 *	<0,0001 *	<0,0001 *	<0,0001 *	<0,0001 *
**TRPML2 ^{low}<br>TRPML1 ^{neg}**	0,0219 *		0,2334	0,576	0,0088 *	0,0004 *
**TRPML2 ^{high}<br>TRPML1 ^{high}**	<0,0001 *	0,2334		0,0259 *	0,0942	<0,0001 *
**TRPML2 ^{high}<br>TRPML1 ^{low}**	<0,0001 *	0,576	0,0259 *		0,0019 *	<0,0001 *
**TRPML2 ^{high}<br>TRPML1 ^{neg}**	<0,0001 *	0,0088 *	0,0942	0,0019 *		<0,0001 *
**TRPML2 ^{neg}<br>TRPML1 ^{neg}**	<0,0001 *	0,0004 *	<0,0001 *	<0,0001 *	<0,0001 *	

Table S4 Pearson's Correlation Coefficient

**Markers**	**PCC<br>T98**	**PCC<br>U251**
TRPML2<br>Calreticulin	0.86	0.82
TRPML2<br>Caveolin 1	0.23	0.26
TRPML2<br>COX IV	0.27	0.29
TRPML2<br>LAMP1	0.81	0.86
TRPML1<br>Calreticulin	0.66	0.64
TRPML1<br>Caveolin 1	0.25	0.19
TRPML1<br>COX IV	0.18	0.20
TRPML1<br>LAMP1	0.67	0.65

0<PCC<0.3= weak positive correlation; 0.3<PCC<0.5= moderate positive correlation; >0.05 strong positive correlation. PCC= Pearson's Correlation Coefficient
